# Prognostic Value of Polypoid Changes of the Middle Turbinate in Relapsed Nasal Polypi after FESS: A Prospective Cohort Study

**DOI:** 10.1055/s-0043-1776730

**Published:** 2023-11-29

**Authors:** Alaa Mohamed Abdelsamie, Hossam Mohamed Abdelazeem, Gena Kerollos Dawood, Taha Mohamed Abdelaal

**Affiliations:** 1 Department of Otorhinolaryngology, Faculty of Medicine, Benha University, Benha, Egypt

**Keywords:** allergic rhinitis, chronic rhinosinusitis, nasal endoscopy, nasal polyposis, nasal turbinate

## Abstract

**Introduction** Despite the high level of patient satisfaction with functional endoscopic sinus surgery (FESS) and the clinical improvement, polyp recurrence is observed in 23% to 87% of patients and requires reoperation.

**Objective** To assess the prognostic value of polypoid changes of the middle turbinate (PCMT) in relapse of paranasal sinus polyps in patients with chronic rhinosinusitis with nasal polyp (CRSwNP) after FESS and the effect of partial middle turbinectomy (PMT) on the outcome of surgery.

**Methods** We conducted a prospective clinical study on 60 patients with CRSwNP with and without PCMT. The patients were allocated into three groups: group I included twenty patients without PCMT; group II, twenty patients with PCMT; and group III included twenty patients with PCMT submitted to PMT. The patients were evaluated endoscopically according to the Lund-Kennedy endoscopic scoring system, radiologically according to the Lund-Mackay scoring system, and symptomatically through the 22-item Sinonasal Outcome Test (SNOT-22).

**Results** The total postoperative Lund-Kennedy score differed significantly among the 3 groups ( *p* < 0.001), with a group II presenting a significantly higher total score compared to groups I and III. The Preoperative SNOT-22 score differed significantly among the three groups ( *p* = 0.013), with group II presenting a significantly higher score compared to group I. There was a significant association involving the 3 groups and relapse at 12 months ( *p* = 0.029); relapse was higher in group II (50.0%) than in groups I (20%) and III (15.0%).

**Conclusion** There was a significant association between PCMT and the relapse of nasal polyps. Also, nasal polyposis recurred at a lower rate in the group submitted to middle turbinate resection compared to the group in whom it was preserved.

## Introduction

Chronic rhinosinusitis (CRS) is a common inflammatory disorder that may present with nasal polyps (CRSwNP), which have been found to occur in 4% of the general population. 1

Currently, the main treatment for nasal polyps that do not respond to medical therapy is surgery. Functional endoscopic sinus surgery (FESS) is characterized by the restoration of the mucociliary function of the nasal cavity and paranasal sinuses, and it enables the administration of anti-inflammatory drugs in the surgical cavity.

Despite the high level of patient satisfaction with FESS and the clinical improvement, polyp recurrence is observed in 23% to 87% of the patients, and it requires reoperation. 2

Many studies 3 on the risk of recurrence in CRSwNP patients who underwent FESS found that nonsteroidal anti-inflammatory drugs (NSAIDs) exacerbated the respiratory disease, and that higher preoperative total scores and frontal sinus computed tomography (CT) scores were potentially prognostic.

Despite undergoing FESS and continued medical therapy, 40% of the patients after 18 months of follow-up present polyp recurrence which is comparable to or better than other cohort studies that report recurrence rates ranging from 50% to 60%. 4 5

In an examination of the association between the extent of the surgery and polyp recurrence in a previous single-center cohort study, the authors 6 found that more extensive surgery was protective against polyp recurrence, reporting that nasalization of the ethmoids (that is, complete mucosal stripping coupled with complete marsupialization) was associated with lower rates of polyp recurrence compared with “functional” ethmoidectomy.

In the literature, 4 there are reports of an association between Draf III frontal sinusotomy and reduced prevalence of polyp recurrence. Moreover, it has been suggested that polypoid changes of the middle turbinate (PCMT) anterior free border is a unique prognostic. 7 Partial middle turbinectomy (PMT) has been shown to enable the administration of drugs to the frontal and sphenoidal sinuses after the operation. Therefore, it helps to decrease the development of polypoidal changes. 1

The present study aimed to assess the prognostic value of PCMT in the relapse of paranasal sinus polyps in patients with CRSwNP and the potential influence of PMT on the outcome of surgery.

## Materials and Methods

We performed a prospective clinical study at the Otolaryngology and Head and Neck Surgery Department of our institution involving 60 CRSwNP adult patients with and without PCMT.

### Inclusion and Exclusion Criteria

Adult patients with CRS symptoms persisting for more than three months, endoscopic evidence of polyps, and/or mucosal changes evidence on CT who showed no response to the standard therapy regimes were candidates for FESS.

Patients with congenital mucociliary disorders, cystic fibrosis, aspirin hypersensitivity, allergic fungal rhinosinusitis, bronchial asthma, systemic vasculitis, immune deficiency, and those with a history of sinus surgery were excluded.

### Intervention

**Fig. 1:**
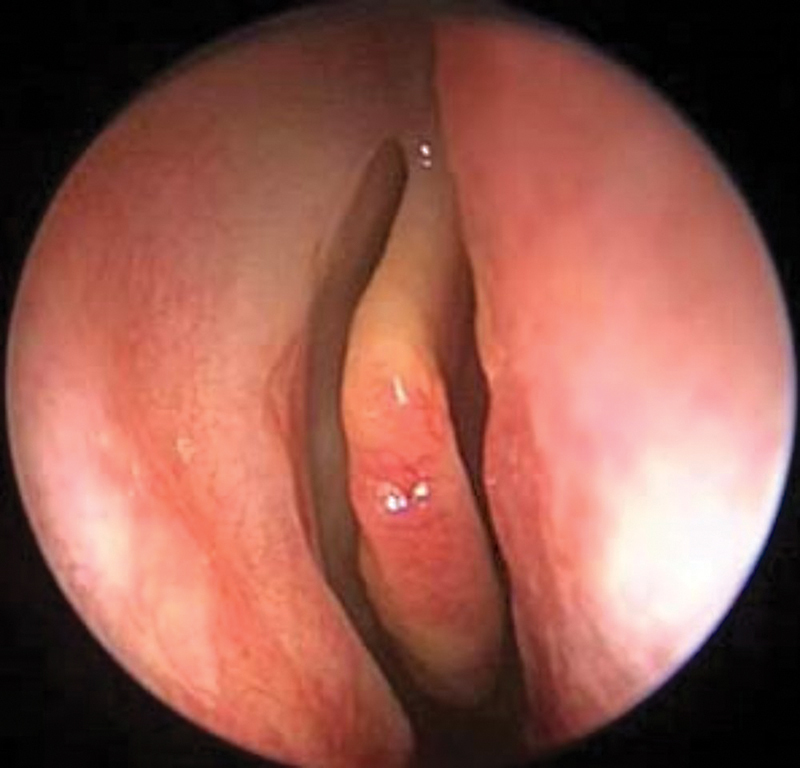
Middle turbinate without polypoid change.

**Fig. 2:**
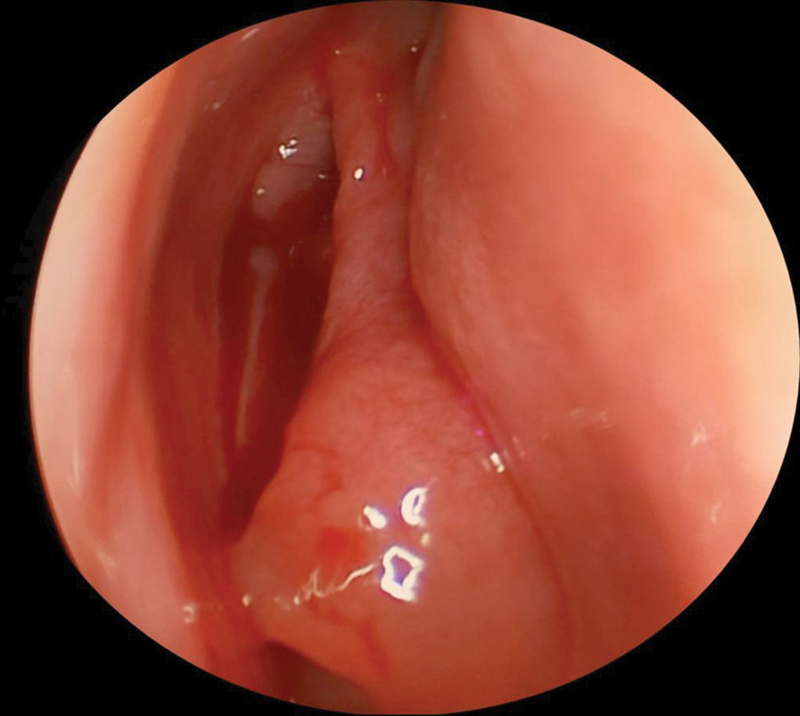
Middle turbinate with polypoid change.

The study sample was composed of three groups of CRSwNP patients who underwent endoscopic sinus surgery as follows:

- Group I: twenty patients without PCMT ( Fig. 1 ).
- Group II: twenty patients with PCMT ( Fig. 2 ).
- Group III: twenty patients with PCMT submitted to PMT.

Endoscopic grading was based on Lund-Kennedy endoscopic scoring system, and polypoid changes were defined as mucosal edema or changes that involve anterior free border of the turbinate. A paranasal sinus CT examination was performed, and the patients were scored based on the Lund-Mackay scoring system. The 22-item Sinonasal Outcome Test (SNOT-22) was used to assess symptomatology and health-related quality of life.

After identifying the middle turbinate (MT), which is the principal landmark for the procedure, the uncinated process was located on the lateral nasal wall at the level of the anterior end of the MT, and it was resected, with subsequent exposure of the hiatus semilunaris and the ethmoidal bulla.

The anterior ethmoidal air cells were opened to enable proper aeration while keeping the mucosa over the bone. An inspection of the maxillary ostium was then performed. If the ostium was obstructed, it was opened through a middle meatus antrostomy. Usually, such a minimal surgical procedure would be adequate to provide great improvement in ostiomeatal complex (OMC) function, with consequently better aeration of the maxillary, ethmoidal, and frontal sinuses.

**Fig. 3:**
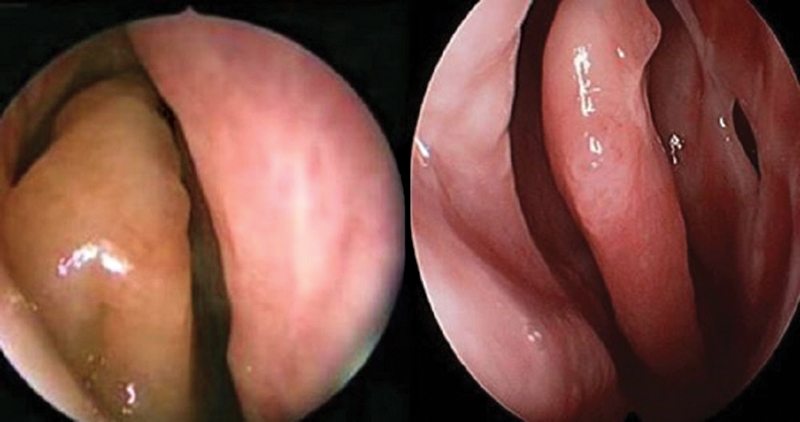
Polypoid middle turbinate before and after partial resection

When the CT scans showed diseased posterior ethmoidal and sphenoidal sinuses, it was essential to proceed into these sinuses. At the end of the procedure, full FESS using the standard technique was performed for groups I and II, with additional PMT for group III ( Fig. 3 ).

### Pre- and Postoperative Treatment Regimen

Patients with severe polyposis or markedly hyperreactive mucosa were best treated with a course of oral steroids to reduce mucosal bleeding, unless there was a contraindication to their use; 20 to 30 mg of prednisone for 3 to 10 days preoperatively were sufficient.

Infection should be minimized, if possible, to reduce intraoperative bleeding. In severe cases of the disease, this may require a culture-directed antibiotic course of two or more weeks. Nasal saline irrigation, sinus cavity debridement, and standard topical nasal steroid spray (mometasone furoate) were applied postoperatively.

Postoperatively, all patients were subjected to nasal endoscopy, and grading was performed based on the Lund-Kennedy endoscopic scoring system and documented at 4 weeks, 6 months, and 1 year. The score is as follows: 0–absence of polyps; 1– polyps only in the middle meatus; and 2–polyps beyond the middle meatus. The presence of a polyp for one year during endoscopy was defined as polyp relapse. The SNOT-22 scores were recorded at the 1-year visit.

### Statistical Analysis

Data management and statistical analysis were performed using the IBM SPSS Statistics for Windows (IBM Corp., Armonk, NY, United States) software, version 25.0. Quantitative data were assessed for normality using the Shapiro-Wilk test and direct data visualization methods. According to the normality test, numerical data were expressed as mean and standard deviation values or medians and ranges. Categorical data were expressed as numbers and percentages. The quantitative data were compared among the study groups using one-way analysis of variance (ANOVA) or the Kruskal-Wallis test for normally and non-normally distributed numerical variables respectively. Categorical data were compared using the Chi-squared test. All post hoc analyses were adjusted through the Bonferroni correction. All statistical tests were two-sided. Values of *p* lower than 0.05 were considered statistically significant.

## Results

### General Characteristics

No significant differences were observed among the three groups in terms of age ( *p* = 0.841) and gender ( *p* = 0.626) ( Table 1 ).

**Table 1:** Age and gender distribution of the study groups

		Group I ( *n* = 20)	Group II ( *n* = 20)	Group III ( *n* = 20)	*p* -value
**Age (in years): mean ± standard deviation**		37 ± 12	38 ± 13	39 ± 8	0.841
**Gender: male/female – n (%)**		12 (60.0)/8 (40.0)	11 (55.0)/9 (45.0)	9 (45.0)/11 (55.0	0.626
				)	

Notes: One-way analysis of variance (ANOVA) was used for age, and the Chi-squared test was used for gender.

### Preoperative Lund-MacKay Score (Radiological Staging)

No significant differences were observed among the study groups in the preoperative Lund-MacKay score for the following: maxillary paranasal sinus ( *p* = 0.579), anterior ethmoid paranasal sinus ( *p* = 0.355), posterior ethmoid paranasal sinus ( *p* = 0.125), sphenoid paranasal sinus ( *p* = 0.426), frontal paranasal sinus ( *p* = 0.426), OMC ( *p* = 0.368), and total score ( *p* = 0.334) ( Table 2 ).

**Table 2:** Preoperative Lund-MacKay score

		Group I ( *n* = 20)	Group II ( *n* = 20)	Group III ( *n* = 20)	*p* -value
**Maxillary: median (range)**		2 (1–2)	2 (1–2)	2 (1–2)	0.579
**Anterior ethmoid: median (range)**		2 (1–2)	2 (1–2)	2 (2–2)	0.355
**Posterior ethmoid: median (range)**		2 (1–2)	2 (1–2)	2 (1–2)	0.125
**Sphenoid: median (range)**		1 (1–2)	2 (1–2)	2 (1–2)	0.426
**Frontal: median (range)**		2 (1–2)	2 (1–2)	1 (1–2)	0.426
**Ostiomeatal complex: median (range)**		2 (0–2)	2 (2–2)	2 (2–2)	0.368
**Total: median (range)**		10 (5–12)	11 (9–12)	11 (10–12)	0.334

Note: The Kruskal-Wallis test was used.

### Preoperative Lund-Kennedy Score (Endoscopic Staging)

Table 3 showed that no significant differences were observed among the study groups in the preoperative Lund-Kennedy score regarding polyp ( *p* = 1.0), edema ( *p* = 1.0), discharge ( *p* = 1.0), crusting ( *p* = 0.158), and total score ( *p* = 0.076).

**Table 3:** Preoperative Lund-Kennedy score

		Group I ( *n* = 20)	Group II ( *n* = 20)	Group III ( *n* = 20)	*p* -value
**Polyp: median (range)**		2 (2–2)	2 (2–2)	2 (2–2)	1.0
**Edema: median (range)**		2 (2–2)	2 (2–2)	2 (2–2)	1.0
**Discharge: median (range)**		2 (2–2)	2 (2–2)	2 (2–2)	1.0
**Crusting: median (range)**		2 (1–2)	1 (1–2)	2 (1–2)	0.158
**Total: median (range)**		8 (7–8)	7 (7–8)	8 (7–8)	0.076

Note: The Kruskal-Wallis test was used.

### Preoperative SNOT-22 Score and Origin of Polypi

No significant differences were noted among the three groups regarding the pre-operative SNOT-22 score and the origin of polypi of the MT ( *p* = 0.985) ( Table 4 ).

**Table 4:** Preoperative SNOT-22 score and origin of polypi

			Group I ( *n* = 20)	Group II ( *n* = 20)	Group III ( *n* = 20)	*p* -value
**Preoperative SNOT-22 score: median (range)**			76 (70–81)	79 (75–81)	78 (75–82)	0.118
**Origin of middle turbinate polypi: n (%)**						0.985
*Anterior border*			9 (45.0)	9 (45.0)	9 (45.0)	
*Lateral border*			2 (10.0)	4 (20.0)	4 (20.0)	
*Medial border*			4 (20.0)	3 (15.0)	3 (15.0)	
*Posterior border*			5 (25.0)	4 (20.0)	4 (20.0)	

Abbreviation: SNOT-22, 22-item Sinonasal Outcome Test.

Note: The Kruskal-Wallis test was used for the SNOT-22. The origin of polypi were compared using the Fisher exact test.

### Postoperative Lund-Kennedy Score at 1 Month

At 1 month, the polyp score differed significantly among the three groups ( *p* < 0.001). Paired comparisons using the post hoc test revealed a significantly higher polyp score in groups I and II (score of 1: polyp limited to the middle meatus) than in group III (score of 0: no polyp). The edema score also differed significantly between the three groups ( *p* < 0.001), with a significantly higher edema score in groups I and II than in group III (0.0). In addition, the discharge score differed significantly among the three groups ( *p* < 0.001), with groups I and II scoring significantly higher than group III. Furthermore, the crusting score also differed significantly between the three groups ( *p* < 0.001), with significantly higher scores in group III compared to the other groups (0). On the other hand, no significant difference was reported among the study groups regarding the total Lund-Kennedy score ( *p* = 0.087) ( Table 5 ).

**Table 5:** Postoperative Lund-Kennedy score at 1 month

		Group I ( *n* = 20)	Group II ( *n* = 20)	Group III ( *n* = 20)	*p* -value
**Polyp: median (range)**		1 (0–1) ^a^	1 (0–2) ^a^	0 (0–0) ^b^	**< 0.001**
**Edema: median (range)**		1 (1–2) ^a^	1 (1–2) ^a^	0 (0–0) ^b^	**< 0.001**
**Discharge: median (range)**		1 (1–1) ^a^	1 (1–1) ^a^	1 (0–1) ^b^	**< 0.001**
**Crusting: median (range)**		0 (0–1) ^a^	0 (0–1) ^a^	2 (1–2) ^b^	**< 0.001**
**Total: median (range)**		3 (2–4)	3 (2–4)	3 (2–3)	0.087

Notes: The Kruskal-Wallis test was used. The post hoc analysis through Bonferroni correction was performed in case of significant overall effect, and different letters (a, b) indicate significant pairs.

### Postoperative Lund-Kennedy Score at 6 Months

**Fig. 4:**
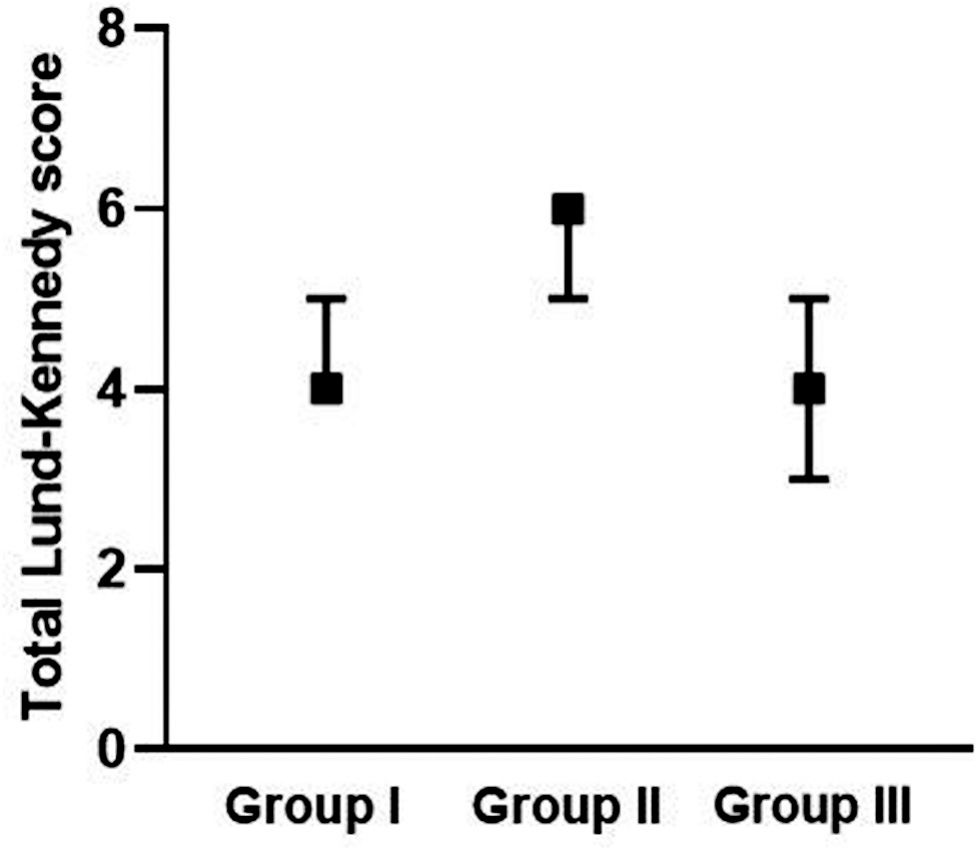
Total postoperative Lund-Kennedy score of the study groups at 6 months.

At 6 months, the polyp score differed significantly among the three groups ( *p* = 0.022), with a significantly higher polyp score range in group II than III. The edema and discharge scores, as well as the total score, also differed ( *p* < 0.001 for the 3 scores): they were significantly higher in group II than in groups I and III. In contrast, no significant difference among the three groups was observed regarding crusting score ( *p* = 0.194) ( Table 6 and Fig. 4 ).

**Table 6:** Postoperative Lund-Kennedy score at 6 months

		Group I ( *n* = 20)	Group II ( *n* = 20)	Group III ( *n* = 20)	*p* -value
**Polyp: median (range)**		1 (1–2) ^a,b^	1 (1–2) ^a^	1 (1–1) ^b^	**0.022**
**Edema: median (range)**		1 (1–2) ^a^	2 (1–2) ^b^	1 (1–2) ^a^	**< 0.001**
**Discharge: median (range)**		1 (1–2) ^a^	2 (1–2) ^b^	1 (1–1) ^a^	**< 0.001**
**Crusting: median (range)**		1 (0–1)	1 (0–1)	1 (0–1)	0.194
**Total: median (range)**		4 (4–5) ^a^	6 (5–6) ^b^	4 (3–5) ^a^	**< 0.001**

Notes: The Kruskal-Wallis test was used. The post hoc analysis through Bonferroni correction was performed in case of significant overall effect, and different letters (a, b) indicate significant pairs.

### Post-operative Lund-Kennedy Score at 12 Months

**Fig. 5:**
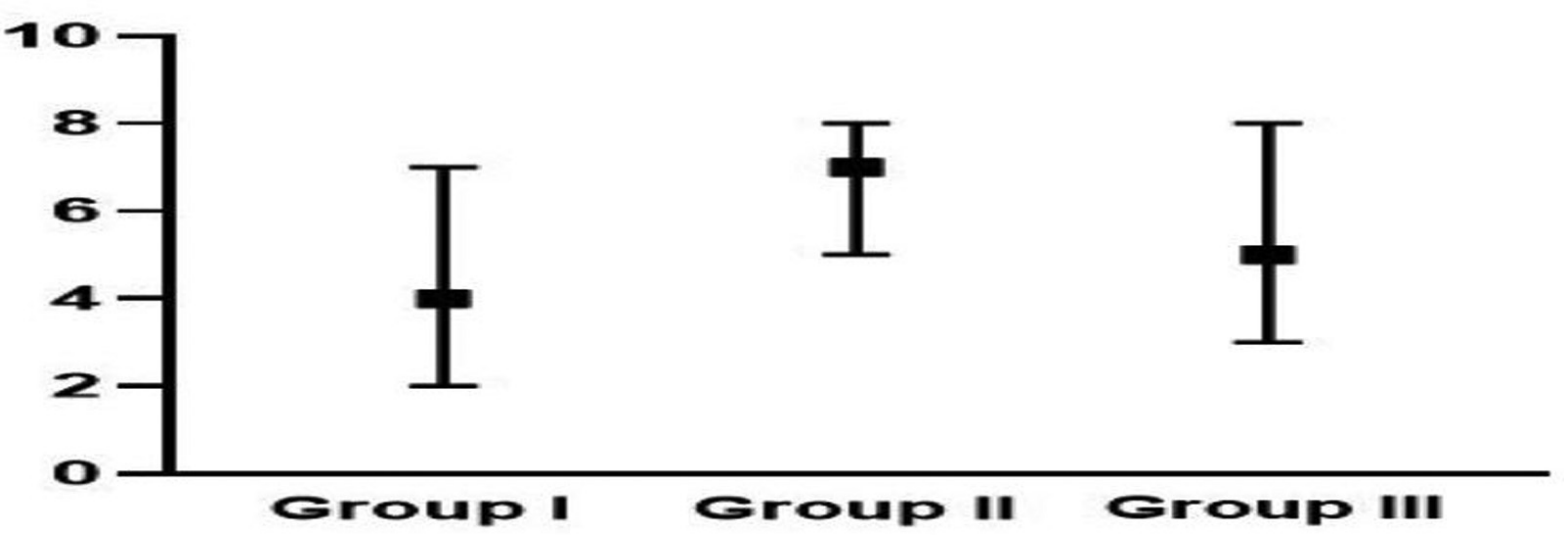
Postoperative Lund-Kennedy score of the study groups at 12 months .

At 12 months, the polyp score differed among the three groups ( *p* = 0.025),: it was significantly higher in group II (score of 2: polyp extended in the nasal cavity) than in group III (score of 1: polyp limited to the middle meatus). The edema score also differed among the three groups ( *p* = 0.002): it was significantly higher in group II than group III. In addition, the discharge and total scores ( *p* < 0.001 for both) also differed: they were significantly higher in group II than in groups I and III ( Table 7 and Fig. 5 ). In contrast, no significant difference among the three groups ws observed regarding the crusting score ( *p* = 0.104) ( Table 8 ).

**Table 7:** Postoperative Lund-Kennedy score at 12 months

		Group I ( *n* = 20)	Group II ( *n* = 20)	Group III ( *n* = 20)	*p* -value
**Polyp: median (range)**		1 (0–2) ^a,b^	2 (0–2) ^a^	1 (0–2) ^b^	**0.025**
**Edema: median (range)**		2 (1–2) ^a,b^	2 (1–2) ^a^	1 (1–2) ^b^	**0.002**
**Discharge: median (range)**		1 (1–2) ^a^	2 (1–2) ^b^	1 (1–2) ^a^	**< 0.001**
**Crusting: median (range)**		1 (0–1)	1 (0–2)	1 (1–2)	0.104
**Total: median (range)**		4 (2–7) ^a^	7 (5–8) ^b^	5 (3–8) ^a^	**< 0.001**

Notes: The Kruskal-Wallis test was used. The post hoc analysis through Bonferroni correction was performed in case of significant overall effect, and different letters (a, b) indicate significant pairs.

**Table 8:** Postoperative Lund-Kennedy score

		Group I ( *n* = 20)	Group II ( *n* = 20)	Group III ( *n* = 20)	*p* -value
**At 1 month: median (range)**		3 (2–4)	3 (2–4)	3 (2–3)	
					0.087
**At 6 months: median (range)**		4 (4–5) ^a^	6 (5–6) ^b^	4 (3–5) ^a^	
					**< 0.001**
**At 12 months: median (range)**		4 (2–7) ^a^	6 (5–8) ^b^	5 (3–8) ^a^	
					**< 0.001**

Notes: The Kruskal-Wallis test was used. The post hoc analysis was performed through Bonferroni correction and different letters (a, b) indicate significant pairs.

### Pre- and Postoperative SNOT-22 Score

The preoperative SNOT-22 score differed among the three groups ( *p* = 0.013), being significantly higher in group II (score of 80) than in group I (score of 76). The postoperative SNOT-22 score also differed ( *p* < 0.001), being significantly higher group II (score of 45) than in groups I and III. The changes in percentages also differed ( *p* < 0.001): they were significantly lower in group II (-43.7%) compared to groups I (-90.7%) and III (-90.9%) ( Table 9 ).

**Table 9:** Pre- and postoperative SNOT-22 score

		Group I ( *n* = 20)	Group II ( *n* = 20)	Group III ( *n* = 20)	*p* -value
**Preoperative: median (range)**		76 (70–81) ^a^	80 (75–81) ^b^	78 (75–82) ^a,b^	**0.013**
**Postoperative: median (range)**		7 (6–70) ^a^	45 (10–80) ^b^	7 (6–77) ^a^	**< 0.001**
**Percentage change: median (range)**		-90.7 (-92.6–-6.8) ^a^	-43.7 (-87.7–-2.6) ^b^	-90.9 (-92.4–-4.9) ^a^	**< 0.001**

Abbreviation: SNOT-22, 22-item Sinonasal Outcome Test.

Notes: The Kruskal-Wallis test was used. The post hoc analysis through Bonferroni correction was performed in case of significant overall effect, and different letters (a, b) indicate significant pairs.

### Relapse

There was a significant association involving relapse at 12 months and the studied groups ( *p* = 0.029): it was higher in group II (50.0%) than in groups I (20%) and III (15.0%) ( Table 10 ).

**Table 10:** Relapse at 12 months

		Group I ( *n* = 20)	Group II ( *n* = 20)	Group III ( *n* = 20)	*p* -value
**Relapse at 12 months: n (%)**		4 (20.0)	10 (50.0)	3 (15.0)	**0.029**

Note: The Chi-squared test was used.

## Discussion

Recurrence of nasal polyposis after treatment is a frequent event, with rates ranging from 40% to 90% of the patients.

The current study was performed to assess the potential association of PCMT anterior free border with the risk of relapse in patients with CRSwNP and to investigate the effect of PMT in lowering the recurrence rate of polyposis.

In the present study, no significant differences were observed among the groups in terms of age and gender, which is in line with In a randomized controlled study with 154 patients conducted by Amali et al. 7

Lund and Kennedy 8 reported that the scores obtained by endoscopy are based on the existence of polyps, which explains the higher endoscopy scores in patients with polyps. This is in line with our preoperative evaluation.

In the present study, the preoperative SNOT-22 score (clinical evaluation) showed an overall significant difference: it was higher in group II than in group I.

Składzeń et al. 9 for the higher SNOT-22 scores among patients with nasal polyps: firstly, they are predisposed to the mass effect of the polyps, which leads to more obstruction of the nasal airway; secondly, they present more viscid postnasal secretions due to the lack of ciliated surfaces on the polyp, with resultant reduced mucociliary clearance.

Orlandi and Terrell 10 also explained the higher frequency of nasal crusting that occurs in patients with nasal polyps: they present inflammatory mucosal and edematous changes, and this inflammation results in patient discomfort. Moreover, patients with nasal polyps often present disturbed olfactory function, which also leads to higher SNOT-22 scores.

The postoperative improvement in symptoms was mirrored in the decrease in the SNOT-22 scores, which were significantly lower in patients with an apparently normal MT anterior free border and in patients who underwent PMT.

Stewart et al. 11 reported a rate of 85% of symptom improvement in CRS patients, as shown in the SNOT-22 score 1 year after the operation, while the rate among patients with nasal polyps was of 81%. Despite being small, this difference was statistically significant ( *p* = 0.003). However, Marchioni et al. 12 disagreed with this result, and reported that, at the postoperative 6-month follow-up, the quality of life improved in the group in whom the MT was resected and in those patients in whom it was preserved ( *p* < 0.001).

In the present study, the Lund-Kennedy scores that reflect the severity of the postoperative inflammation in the surgical cavity were significantly lower in patients with no PCMT and in patients who underwent PMT. On the endoscopic examination 6 and 12 months postoperatively, the relapse rate was higher in group II than groups I ( *p* = 0.022) and III ( *p* = 0.025), which indicates that PCMT plays a role in the recurrence of polyposis after FESS.

Soler et al. 13 reported that patients with PCMT relapsed at a higher rate than the control group. But Garrel et al. 14 found that the baseline nasal polyp stage did not show correlation with postoperative recurrence or the long-term outcome of the surgery.

Furthermore, in the present study, the total scores showed that the patients who underwent MT resection (group III) and those who did not present PCMT (group I) presented almost equal outcomes, which were better than those of the group with PCMT who did not undergo PMT (group II; *p* < 0.001). In contrast, no significant difference was observed among the studied groups regarding the crusting score ( *p* = 0.104).

Scangas et al. 15 recommended the performance of PMT to improve the intraoperative access, reduce the formation of synechiae, and facilitate the postoperative administration of drugs. This contradicts the studies that support preserving the MT to preclude potential turbinectomy complications, including iatrogenic frontal sinusitis, atrophic rhinitis, and anosmia.

In the present study, relapse was higher in group II (50.0%) than in groups I (20%) and III (15.0%). So, resection of the MT resulted in a decrease in the rate of recurrence of polyposis after FESS.

Halderman et al. 1 reported that PMT provides a route for drug delivery to the frontal and sphenoidal sinuses after the operation, thus facilitating the development of mucosal polyps. Wu et al. 2 reported that, in patients subjected to FESS with PMT, the interval until revision surgery was longer.

Overall, the current study showed a significant association between PCMT and nasal polyp relapse, as well as the role of PMT in patients with CRSwNP.

## Strengths and Limitations

As for the strengths, the present study not only shed light on the presence of PCMT as an important prognostic factor for the recurrence of sinonasal polyposis but also gave the clue of the problem (group III) by its partial resection. After endoscopic sinus surgery, PMT plays a role in decreasing the chance of polyp recurrence. Regarding the limitations, a long follow-up is needed to assess the definite time and accurate rate of recurrence; to get more detailed results, a study with a larger sample is also necessary.

## Conclusion

The present study showed a significant association between PCMT and nasal polyp relapse after FESS. Moreover, the patients submitted to OPMT (group III) presented lower rates of recurrence of nasal polyposis recurrence than the patients not submitted to the procedure (group II) MT. Therefore, in cases of PCMT, the surgeon must keep in mind the high rate of recurrence after surgery. So, close follow-up and mandatory PMT are recommended in such cases to reduce the rate of polyp recurrence.
